# *FDPS* links tumor progression, phosphoproteomic reprogramming, and therapeutic vulnerability in hepatocellular carcinoma

**DOI:** 10.17305/bb.2026.13996

**Published:** 2026-04-14

**Authors:** Hanxun Yue, Guozhi Wu, Na Jiang, Renpeng Li, Ya Zheng, Yuping Wang, Zenan Hu, Yongning Zhou

**Affiliations:** 1 The First School of Clinical Medicine, Lanzhou University, Lanzhou, China; 2 Department of Gastroenterology, The First Hospital of Lanzhou University, Lanzhou, China; 3 Gansu Province Clinical Research Center for Digestive Diseases, The First Hospital of Lanzhou University, Lanzhou, China; 4 School of Public Health, Lanzhou University, Lanzhou, China

**Keywords:** Hepatocellular carcinoma, HCC, farnesyl diphosphate synthase, *FDPS*, phosphoproteomics, Caspase-9/3, mTOR signaling, pamidronate

## Abstract

Primary liver cancer is a leading cause of cancer-related death worldwide, and hepatocellular carcinoma (HCC) accounts for most cases. This study aimed to clarify the expression pattern, biological role, therapeutic relevance, and phosphoproteomic impact of farnesyl diphosphate synthase (*FDPS*) in HCC. *FDPS* expression was assessed in public datasets and clinical specimens using quantitative real-time polymerase chain reaction and immunohistochemistry. Its function was investigated through small interfering RNA-mediated silencing, lentiviral overexpression, pamidronate treatment, *in vitro* proliferation, migration, invasion, and apoptosis assays, xenograft models, phosphoproteomic profiling, and Western blot validation. *FDPS* was upregulated in HCC tissues and was potentially associated with poorer survival. *FDPS* silencing reduced HCC cell proliferation, migration, invasion, and tumorigenicity and increased apoptosis, whereas *FDPS* overexpression produced opposite effects. Pamidronate, a selective FDPS inhibitor, suppressed malignant phenotypes *in vitro* and inhibited tumor growth *in vivo*. Phosphoproteomic analysis revealed extensive phosphorylation changes after *FDPS* depletion, with enrichment in pathways related to GTPase and protein kinase C activation, glucose metabolism, cytoskeletal remodeling, immune regulation, cell cycle control, Golgi function, and ErbB and phospholipase D signaling. *FDPS* knockdown was further associated with decreased B-cell lymphoma 2 expression, reduced phosphorylation of Caspase-9 at Ser196, increased cleaved Caspase-3, and reduced phosphorylation of mechanistic target of rapamycin at Ser2448. Collectively, these findings suggest that *FDPS* contributes to HCC progression and may serve as a candidate biomarker and therapeutic target.

## Introduction

Farnesyl diphosphate synthase (FDPS), also known as FPS, FPPS, or PRORK9, is a crucial enzyme in the mevalonate pathway. It catalyzes the synthesis of farnesyl pyrophosphate from isopentenyl diphosphate and geranyl diphosphate [1]. The *FDPS* gene, according to the National Center for Biotechnology Information (NCBI) Gene database, is located on chromosome 1q22 and consists of 11 exons and 8 transcript variants [2]. *FDPS* plays a pivotal role in cellular metabolism and is involved in various physiological and pathophysiological processes, including protein phosphorylation and membrane biosynthesis [3].

Initial investigations into *FDPS* concentrated on its role in bone metabolism, revealing that elevated *FDPS* expression significantly reduces bone mineral density and contributes to osteoporosis progression [4–6]. Subsequent research established that mutations in *FDPS* are a key pathogenic factor in disseminated superficial actinic porokeratosis [7, 8]. Increasing evidence suggests that *FDPS* promotes the progression of several malignancies, including prostate cancer, lung cancer, hypopharyngeal carcinoma, and glioblastoma [9–12]. Over the past decade, the oncogenic role of *FDPS* has attracted substantial scientific interest, with research exploring its potential as both a diagnostic biomarker and a therapeutic target (Table 1). Todenhöfer et al. first identified *FDPS* overexpression as an independent risk factor for early recurrence following chemotherapy in prostate cancer [13]. Following this, Long et al. developed a gene panel that includes *FDPS*, demonstrating its utility in the molecular diagnosis of prostate cancer [14]. The expression level of *FDPS* has been shown to correlate significantly with tumor, node, metastasis (TNM) staging in non-small cell lung cancer (NSCLC). Mechanistically, *FDPS* mediates transforming growth factor beta 1 (TGF-β1)-induced cellular invasion and epithelial-mesenchymal transition, thereby contributing to NSCLC progression [15]. Recent findings from a mitochondria-related genome-wide Mendelian randomization study have revealed a strong correlation between *FDPS* and breast cancer [16]. Correspondingly, a single-cell transcriptomic analysis demonstrated that elevated *FDPS* expression in breast cancer is associated with increased tumor invasiveness and poorer patient prognosis [17]. Wang et al. reported that *FDPS* enhances cervical cancer cell proliferation by modulating the alternative splicing of genes such as bone morphogenetic protein 1 (*BMP1*), semaphorin 4D (*SEMA4D*), annexin A2 (*ANXA2*), and sirtuin 2 (*SIRT2*), which affects the cell cycle and DNA damage repair [18]. Notably, *FDPS* may also exhibit tumor-suppressive properties; Criscuolo et al. found that reduced *FDPS* expression in ovarian cancer is significantly correlated with unfavorable clinical outcomes and increased chemoresistance [19].

**Table 1:** Representative studies of *FDPS* in cancer research

**First author**	**Year**	**Origin**	**Related molecules/pathways**	**Function**	**Reference**
Lin	2012	Bone	ERK	Oncogenesis	[43]
Li	2023	Breast	–	Oncogenesis	[16]
Vishnubalaji	2023	Breast	–	Oncogenesis	[17]
Syed	2023	Breast	–	Oncogenesis	[44]
Wang	2023	Cervix	*BMP1, SEMA4D, ANXA2, SIRT2*	Oncogenesis	[18]
Fiore	2019	Colorectum	*FBXW7*, SREBP	Oncogenesis	[45]
Seo	2020	Colorectum	–	Oncogenesis	[46]
Gao	2021	Colorectum	*HMGCR*, TGF-β1	Oncogenesis	[47]
Lin	2018	Lung	TGF-β1	Oncogenesis	[15]
Criscuolo	2020	Ovary	*TRAP1*	Tumor suppression	[19]
Han	2019	Pancreas	–	Oncogenesis	[48]
Todenhöfer	2013	Prostate	–	Oncogenesis	[13]
Long	2014	Prostate	–	Oncogenesis	[14]
Qiu	2017	Stomach	–	Oncogenesis	[49]

**Abbreviations:** *ANXA2*, annexin A2; *BMP1*, bone morphogenetic protein 1; ERK, extracellular signal-regulated kinase; *FBXW7*, F-box and WD repeat domain containing 7; *FDPS*, farnesyl diphosphate synthase; *HMGCR*, 3-hydroxy-3-methylglutaryl-coenzyme A reductase; *SEMA4D*, semaphorin 4D; *SIRT2*, sirtuin 2; SREBP, sterol regulatory element-binding protein; TGF-β1, transforming growth factor beta 1; *TRAP1*, TNF receptor-associated protein 1.

Nitrogen-containing bisphosphonates (N-BPs) act as specific inhibitors of *FDPS*. Currently, N-BPs, including pamidronate, alendronate, risedronate, ibandronate, minodronate, and zoledronate, are widely used in clinical practice for the treatment of osteoporosis [20]. We hypothesize that N-BPs may inhibit the progression of malignancies by selectively targeting *FDPS* activity. However, this hypothesis requires further validation.

The role of *FDPS* in tumorigenesis remains inadequately understood. While preliminary studies have suggested the potential of *FDPS* as a diagnostic biomarker in hepatocellular carcinoma (HCC), its regulatory function in HCC has yet to be elucidated. In this study, *FDPS* was analyzed using the Gene Expression Profiling Interactive Analysis (GEPIA) database. The expression profile of *FDPS* was validated through quantitative real-time PCR (qRT-PCR) and immunohistochemistry. *FDPS* was genetically and pharmacologically inhibited, while its expression was upregulated using lentivirus in HCC cell lines. The proliferation, migration, invasion, apoptosis, and tumorigenicity of HCC cell lines were subsequently assessed. Phosphoproteomic analysis was conducted to investigate the influence of *FDPS* on the phosphorylation profile of HCC cells. Additionally, the impact of *FDPS* on apoptosis pathways and mechanistic target of rapamycin (mTOR) signaling in HCC was explored (Figure 1).

**Figure 1.**
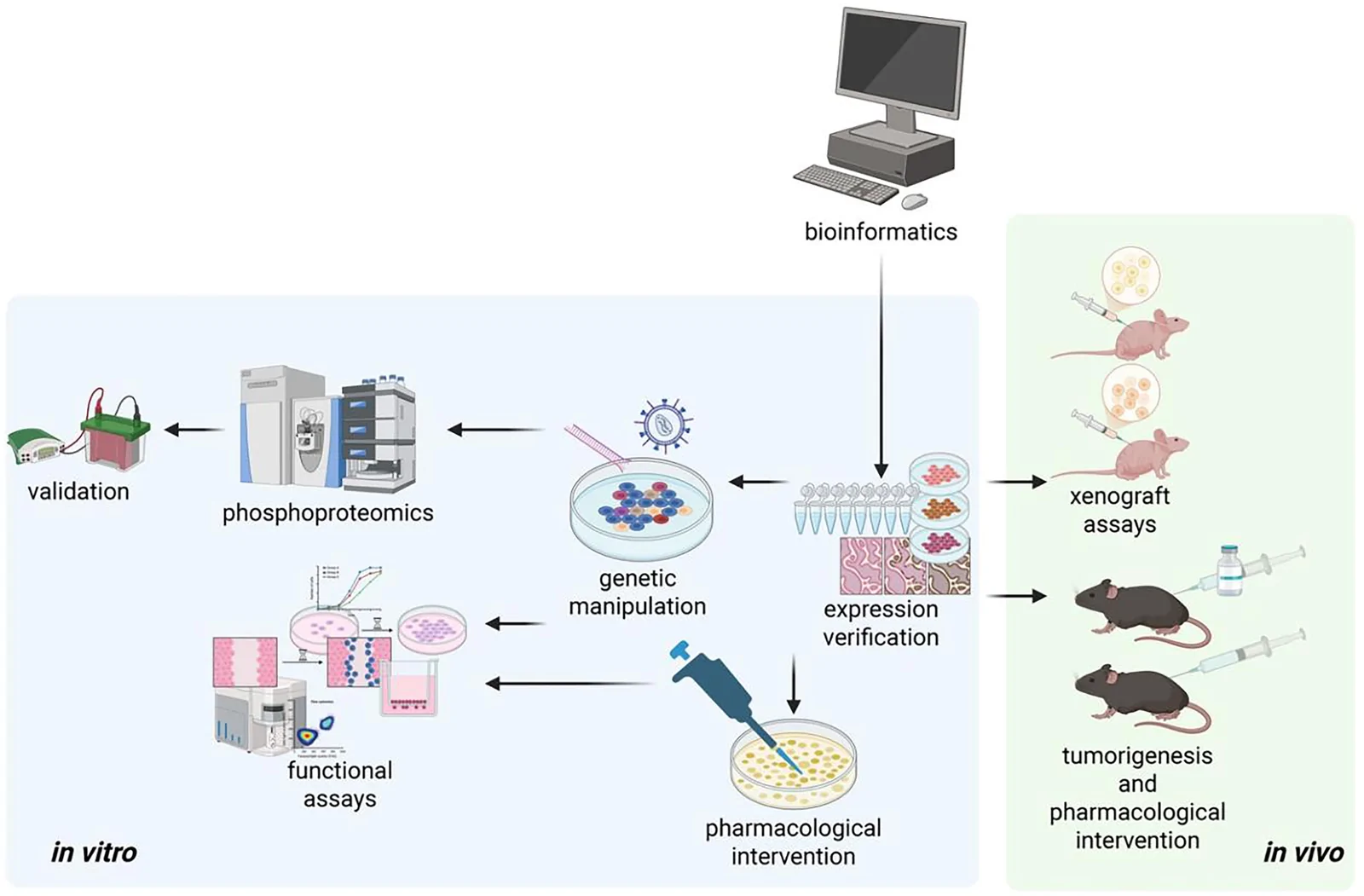
**Schematic overview of the study workflow.** *FDPS* expression in HCC was first assessed by bioinformatic analysis using GEPIA and then verified experimentally in clinical specimens and cell lines by qRT-PCR and immunohistochemistry. After expression verification, *FDPS* was modulated *in vitro* by siRNA-mediated knockdown, lentiviral overexpression, and pharmacological intervention, followed by functional assays evaluating proliferation, migration, invasion, and apoptosis, as well as phosphoproteomic profiling and downstream validation of selected apoptosis-related changes and mTOR signaling. In parallel, *in vivo* xenograft assays and pharmacological intervention models were used to assess tumorigenicity and the antitumor effects of *FDPS* targeting. **Abbreviations:** *FDPS*, farnesyl diphosphate synthase; GEPIA, Gene Expression Profiling Interactive Analysis; HCC, hepatocellular carcinoma; mTOR, mechanistic target of rapamycin; qRT-PCR, quantitative real-time polymerase chain reaction; siRNA, small interfering RNA.

## Materials and methods

### GEPIA analysis

Expression and survival analyses of *FDPS* were conducted using the GEPIA database (http://www.gepia.cancer-pku.cn), which integrates data from the Cancer Genome Atlas (TCGA) and Genotype-Tissue Expression (GTEx) projects. Differential expression was assessed using analysis of variance (ANOVA), with a |log_2_ fold change| threshold of 1 and a significance cutoff of *P* < 0.01. For survival analysis, patients were stratified into high- and low-*FDPS* groups using the median *FDPS* expression level as the cutoff.

### Cell culture and samples

Human HCC cell lines (Huh-7, PLC/PRF/5, and HCCLM3) were obtained from Pricella (Wuhan, China), while the human normal liver cell line THLE-2 and mouse HCC cell line Hepa1-6 cells were acquired from Immocell (Xiamen, China). Authentication of cell lines was conducted by Biowing (Shanghai, China). All cell lines were cultured in Dulbecco’s Modified Eagle Medium (DMEM; Solarbio, Beijing, China) supplemented with 10% fetal bovine serum (FBS; Solarbio) and 1% penicillin-streptomycin (Beyotime, Shanghai, China), and maintained at 37^∘^C in a humidified incubator (Thermo Fisher Scientific, Waltham, MA, USA) containing 5% CO_2_.

Complementary DNA (cDNA) samples and formalin-fixed, paraffin-embedded sections of HCC and adjacent normal tissues were collected from the Department of Gastroenterology, the First Hospital of Lanzhou University. In the anonymized archival records of tissue sections, overall survival was defined as the interval from the date of surgery to the date of death from any cause or the date of last follow-up. Patients who remained alive at the end of the follow-up period or were lost to follow-up were considered right-censored.

### RNA manipulation and drugs

Small interfering RNA specifically targeting *FDPS* (siRNA-*FDPS*) and the corresponding negative control (siRNA-NC) were designed and synthesized by GenePharma (Shanghai, China). Three siRNA-*FDPS* sequences were provided for screening (Table S1). Transfection was carried out using Lipofectamine 2000 (Invitrogen, Carlsbad, CA, USA) according to the manufacturer’s instructions. The knockdown of *FDPS* was validated by qRT-PCR and Western blot.

To establish *FDPS*-overexpressing HCC cell lines, a customized lentiviral vector (Ubc-MCS-3FLAG-CBh-gcCFP-IRES-*FDPS*) was purchased from Genechem (Shanghai, China). Transfection efficiency was assessed via fluorescence intensity. Stably transfected cells were selected using puromycin (MCE, Monmouth Junction, NJ, USA), and successful *FDPS* overexpression was confirmed by qRT-PCR and Western blot.

Pamidronate disodium (C_3_H_9_NNa_2_O_7_P_2_) was obtained from MCE and utilized according to the manufacturer’s guidelines.

### Immunohistochemistry

Tissue sections were deparaffinized using an environmentally friendly deparaffinization solution (Servicebio, Wuhan, China) followed by rehydration in anhydrous ethanol (Sinopharm, Beijing, China). Antigen retrieval was performed using Tris-EDTA buffer (Servicebio), and endogenous peroxidase activity was quenched with 3% hydrogen peroxide (Annjet, Dezhou, China). Non-specific binding was blocked with bovine serum albumin (Servicebio). The sections were then incubated with rabbit anti-*FDPS* primary antibody (dilution, 1:100; Cat.No. 16129-1-AP; Proteintech, Wuhan, China), followed by horseradish peroxidase-conjugated anti-rabbit secondary antibody (dilution, 1:200; Cat.No. G1213-100UL; Servicebio). Signal detection was performed using diaminobenzidine (DAB) chromogen (Servicebio) according to the manufacturer’s protocol. Finally, nuclei were counterstained with hematoxylin (Servicebio), and all images were captured using an identical E100 microscope (Nikon, Tokyo, Japan). Staining intensity was quantified using ImageJ software (version 2.14.0). For each section, five representative rectangular regions of interest (ROIs) were randomly selected, avoiding necrotic, hemorrhagic, and non-specific areas. Image analysis was performed in a blinded manner. Color deconvolution was applied to separate the DAB staining. The images were converted to 8-bit grayscale, and the integrated optical density (IOD) and area of each ROI were measured. The average optical density (AOD) was calculated using the formula: AOD = IOD/Area. Background intensity was measured from adjacent unstained regions and subtracted from each ROI measurement. The mean AOD value of the five ROIs was used to represent the staining intensity of each section. All values were normalized to the mean value of the control group and independently verified by two experienced physicians.

### RNA extraction and qRT-PCR

Total RNA was extracted from cells using TransZol reagent (TransGen, Beijing, China) following the manufacturer’s instructions. Cytoplasmic and nucleic RNA fractions were isolated and purified using the EasyPure RNA kit (TransGen). RNA concentration and purity were assessed using a NanoDrop 2000 spectrophotometer (Thermo Fisher Scientific). cDNA was synthesized on a D100 thermal cycler (Novogene, Beijing, China) from purified RNA with the TransScript One-Step gDNA Removal and cDNA Synthesis SuperMix kit (TransGen). PCR was performed on a QIAquant 96 2Plex system (QIAGEN, Hilden, Germany) using TransStart Top Green qPCR SuperMix (TransGen). Target gene mRNA expression levels were quantified using the 2^−Δ^^Ct^ method and normalized to glyceraldehyde-3-phosphate dehydrogenase (*GAPDH*). Each sample was analyzed in triplicate, and all assays were independently repeated three times. The primer sequences and qRT-PCR cycling conditions are provided in Table S2.

### Protein extraction and Western blot

Total cellular protein was extracted using radioimmunoprecipitation assay (RIPA) lysis buffer (Solarbio), supplemented with protease and phosphatase inhibitor cocktails (both from Selleck, Sugar Land, TX, USA). Protein concentrations were measured using a bicinchoninic acid (BCA) protein assay kit (Thermo Fisher Scientific).

Equal quantities of protein samples were separated using 10% sodium dodecyl sulfate–polyacrylamide gel electrophoresis (SDS-PAGE, Solarbio) and subsequently transferred to polyvinylidene fluoride (PVDF) membranes (MilliporeSigma, Burlington, MA, USA). The membranes were blocked with skim milk (Beyotime) and incubated overnight at 4^∘^C with primary antibodies against FDPS, B-cell lymphoma 2 (Bcl-2), Caspase-9, cleaved Caspase-3, phospho-Caspase-9 (Ser196), mTOR, phospho-mTOR (Ser2448), β-actin, and α-tubulin. Following this, membranes were incubated with horseradish peroxidase (HRP)-conjugated goat anti-rabbit secondary antibodies (detailed antibody information is provided in Table S3) at room temperature for 1 hour. Protein bands were visualized using an enhanced chemiluminescence (ECL) reagent (Thermo Fisher Scientific) with the GD50202 imaging system (Monad, Suzhou, China) and quantified using ImageJ software (version 2.14.0). All assays were conducted in at least three independent biological replicates.

### Enzyme-linked immunosorbent assay (ELISA)

ELISA was performed using commercially available human farnesyl pyrophosphate (FPP) ELISA kits (Mlbio, Shanghai, China), following the manufacturer’s instructions. After the reaction was terminated, absorbance was measured at 450 nm using the Infinite F Plex microplate reader (TECAN, Männedorf, Switzerland). Each sample was analyzed in triplicate, and all assays were independently repeated three times.

### Liquid chromatography–tandem mass spectrometry (LC-MS/MS)

LC-MS/MS was employed to profile the phosphorylation landscape of HCC cells. After protein extraction and quantification, samples underwent enzymatic digestion using acetone (Sinopharm), ammonium bicarbonate solution (Supelco, Bellefonte, PA, USA), trypsin (Promega, Madison, WI, USA), and Lys-C (Thermo Fisher Scientific). The pH of the solution was adjusted to 2–3 with trifluoroacetic acid (Sinopharm), and the supernatant was collected to determine peptide concentration using a peptide detection kit (Thermo Fisher Scientific). The resulting peptide mixtures were enriched using immobilized metal affinity chromatography materials (J&K Scientific, Beijing, China) and eluted with ammonia solution (Rhawn, Shanghai, China). The eluates were subsequently desalted using C18 ZipTips (Millipore) prior to downstream analysis.

During LC separation, mobile phase A consisted of 0.1% formic acid in water, while mobile phase B consisted of 0.1% formic acid in acetonitrile. Peptide samples (200 ng) were loaded onto an Aurora analytical column (IonOpsticks, Melbourne, Australia; 15 cm × 75 µm, packed with 1.6 µm C18 resin) at 50^∘^C. Chromatographic separation was performed at a flow rate of 300 nL/min over a 60-minute gradient.

Mass spectrometric analysis was conducted using a timsTOF Pro2 mass spectrometer (Bruker, Billerica, MA, USA) in data-dependent acquisition–parallel accumulation serial fragmentation (DDA-PASEF) mode. The total analysis time was 60 min, and data were collected in positive ion mode. The precursor ion scan range was set from 100–1700 m/z, with an ion mobility range of 0.7–1.4 Vs/cm^2^. Ion accumulation and release time were set to 100 ms, achieving nearly 100% ion utilization. The capillary voltage was maintained at 1500 V, with a drying gas flow rate of 3 L/min and a drying temperature of 180^∘^C. PASEF acquisition included 10 MS/MS scans per cycle, with a total cycle time of 1.17 s. The charge state range was set from 0–5, with a dynamic exclusion duration of 0.4 min. The target ion intensity was set at 10,000, and the minimum intensity threshold was established at 2,500. Collision energy increased linearly with ion mobility, ranging from 20 eV at 0.6 Vs/cm^2^ to 59 eV at 1.6 Vs/cm^2^. Quadrupole isolation widths were set at 2 Th for precursor ions with m/z < 700 and 3 Th for those with m/z > 800.

Raw MS data were processed and searched using FragPipe software (version 21.1) within a combined.fasta database containing 82,494 sequences. A maximum of two missed cleavages and three variable modifications were permitted, while decoy and contaminant identifications were excluded from the final output. The primary endpoints were defined as differential phosphorylation sites between *FDPS* knockdown and control groups. The data were log2-transformed and normalized using median centering to minimize systematic variation across samples. Differential phosphosites were identified using a moderated *t*-test implemented in the limma R package. To adjust for multiple testing, Benjamini–Hochberg adjusted *P* values were calculated. Given the limited sample size (*n* ═ 3 per group), candidate phosphosites were prioritized based on *P* < 0.05 and |log2 fold change| > 0.58, with key findings validated through independent experiments.

The subcellular localization of differentially phosphorylated proteins was assessed using the WoLF PSORT tool (https://www.genscript.com/wolf-psort). Conserved motif analysis was conducted using the MEME website (https://meme-suite.org). Gene Ontology (GO) and Kyoto Encyclopedia of Genes and Genomes (KEGG) enrichment analyses were performed using the Metware Cloud platform (https://cloud.metware.cn), while KOG annotation was completed via the ResearchEasy platform (http://www.researcheasy.cn). Protein domain enrichment analysis utilized the InterPro database (https://www.ebi.ac.uk/interpro).

### Cell proliferation analysis

Cell proliferation was evaluated using the Cell Counting Kit-8 (CCK-8; Biosharp, Hefei, China) and colony formation assays. In the CCK-8 assay, 3000 cells were seeded into each well of a 96-well plate containing 100 µL of complete medium. At 0, 24, 48, and 72 h post cell adhesion, 10 µL of CCK-8 reagent was added per well, followed by incubation at 37^∘^C in a 5% CO_2_ incubator for 1 hour. Absorbance at 450 nm was measured using the Infinite F Plex microplate reader.

For colony formation assays, 5000 cells were plated into each well of a 6-well plate and cultured for 14 days. Colonies were fixed with 4% paraformaldehyde (Solarbio) and stained with 0.1% crystal violet (Beyotime). Colony images were captured using an X-T2 digital camera (Fujifilm, Tokyo, Japan) and analyzed using Photoshop (version 23.5.2) and ImageJ software. All assays were conducted in three independent biological replicates, with three replicate wells for each condition.

### Cell migration and invasion analysis

Cell migration was assessed using wound healing assays. Cells were seeded in 6-well plates and cultured at 37^∘^C with 5% CO_2_ until reaching over 90% confluence. A linear scratch was made vertically across each well using a sterile 200 µL pipette tip. Detached cells were removed by washing three times with phosphate-buffered saline (PBS; Solarbio), followed by the addition of serum-free medium. Images were captured at 0, 24, and 48 h using an XD microscope (SOPTOP, Ningbo, China), and wound closure was quantified using ImageJ software. All assays were performed in three independent biological replicates, with six fields analyzed per replicate.

Cell invasion was evaluated using Transwell assays. The upper chambers of Transwell inserts (Corning, Corning, NY, USA) were precoated with Matrigel (1:8 dilution; Corning) and allowed to solidify. A total of 5000 cells suspended in medium containing 5% FBS were seeded into the upper chambers, while the lower chambers were filled with 20% FBS medium as a chemoattractant. After 48 h of incubation at 37^∘^C and 5% CO_2_, non-invading cells were removed, and the invaded cells on the lower surface of the membrane were fixed with 4% paraformaldehyde and stained with 0.1% crystal violet. Images were acquired using an XD microscope (SOPTOP), and quantitative analysis was performed with ImageJ software. All assays were performed in three independent biological replicates, with five fields analyzed per replicate.

### Cell apoptosis analysis

Apoptosis analysis was conducted using a NovoCyte Advanteon flow cytometer (Agilent, Santa Clara, CA, USA) following staining with the Annexin V-APC/PI kit (KeyGEN BioTECH, Nanjing, China) according to the manufacturer’s protocol. Prior to analysis, unstained samples were utilized to adjust fluorescence compensation, eliminating spectral overlap and establishing quadrant gate settings. Annexin V-APC fluorescence (Ex = 633 nm, Em = 660 nm) was detected via the APC-H channel (FL4), while PI red fluorescence (Ex = 488 nm, Em ≥ 630 nm) was detected through the PE-H channel (FL2). All assays were performed in triplicate to ensure reproducibility, with three biological replicates.

### Animal models

For xenograft assays, six-week-old male nude mice were obtained from the Lanzhou Veterinary Research Institute, Chinese Academy of Agricultural Sciences (Lanzhou, China). Mice were randomly assigned to experimental and control groups, with eight mice in each group. Huh-7 cells transduced with siRNA-*FDPS* (experimental group) and Huh-7 cells transduced with siRNA-NC (control group) were suspended in DMEM at a concentration of 1 × 10^6^ cells/mL. A volume of 0.1 mL of the cell suspension was subcutaneously injected into the lower left flank of each mouse. At days 5 and 10 following cell inoculation, mice in the experimental group were intratumorally injected with siRNA-*FDPS* (5 nmol/20 g body weight), while control mice received an equivalent dose of siRNA-NC via the same route (Figure S1).

For tumor formation assays, six-week-old male C57BL/6 mice were obtained and acclimatized. Hepa1-6 cells, suspended in PBS at a concentration of 1 × 10^6^ cells/mL, were subcutaneously injected at a volume of 0.1 mL into the lower left flank of each mouse. The mice were randomly divided into two groups (*n* ═ 10 per group) and intraperitoneally administered either 5 mg/kg pamidronate dissolved in PBS (experimental group) or an equal volume of PBS (control group) on days 7, 11, 15, and 19 post-inoculation (Figure S2).

All mice were housed in a specific pathogen-free facility under controlled environmental conditions (temperature 18–22 ^∘^C, relative humidity 50%–60%, 12 h light/12 h dark cycle), with ad libitum access to food and water. Mice from the experimental and control groups were co-housed in the same cages and identified by ear tags, with cage positions systematically rotated every two days. Tumor volume was measured with a vernier caliper and calculated using the formula: tumor volume = 0.5 × long diameter × (short diameter)^2^. Mice that failed to develop palpable tumors within 5 days after inoculation were excluded from the study. In accordance with institutional animal ethics guidelines, mice were euthanized once tumor volume reached 2000 mm^3^. In survival analyses, animals that died or were euthanized due to tumor size exceeding ethical limits were recorded as events (deaths), while animals that remained alive at the end of the experiment were treated as censored. All assays were performed in triplicate to ensure reproducibility.

### Ethical statement

All procedures involving human tissue or animals were reviewed and approved by the Ethics Committee of the First Hospital of Lanzhou University (LDYYLL-2025-118, Lanzhou, China, March 6, 2025). Informed consent was waived because the samples are archived and fully anonymized.

### Statistical analysis

Statistical analyses were performed using GraphPad Prism software (version 10.2.0) and Statistical Package for the Social Sciences (SPSS) software (version 30.0.0.0). For comparisons between two groups, paired or unpaired Student’s *t*-tests were employed, as appropriate, for data meeting the assumptions of normality and homogeneity of variance. For repeated measures over time, intergroup differences were analyzed using two-way repeated-measures ANOVA. For time-course data with missing values, a mixed-effects model was applied. Survival analysis was conducted using the Kaplan–Meier method, with differences between groups assessed by the log-rank test and further evaluated using Cox regression analysis. All tests were two-sided, and *P* < 0.05 was considered statistically significant.

## Results

### *FDPS* is highly expressed in HCC and potentially associated with poor prognosis

GEPIA analysis indicated that *FDPS* expression was significantly elevated in HCC tissues compared to normal liver tissues. Patients exhibiting high *FDPS* expression demonstrated poorer overall survival (Figure 2A and B). To validate these findings, *FDPS* expression levels in cDNA samples were determined via qRT-PCR. After excluding samples with undetectable *FDPS* or *GAPDH* expression, 26 HCC samples and 30 adjacent normal liver tissue samples were included. An unpaired *t*-test was employed to compare expression levels between the two groups. Additionally, a paired *t*-test was conducted on the 26 matched pairs of HCC and adjacent normal tissue samples derived from the same patient. Both unpaired and paired analyses confirmed that *FDPS* was significantly upregulated in HCC tissues (Figure 2C and D). Subsequently, *FDPS* expression was further evaluated by immunohistochemistry in paraffin-embedded tissue sections. After excluding sections with tissue detachment involving more than 30% of the section area, 22 HCC sections and 21 adjacent normal liver sections were included. Immunohistochemical staining corroborated that *FDPS* expression was significantly higher in HCC tissues than in adjacent normal liver tissues (Figure 2E and F). Using the median *FDPS* immunohistochemical staining intensity quantified by ImageJ software as the cutoff value (median, 1.401; range, 0.030–5.226), HCC sections were categorized into a high *FDPS* expression group (*n* ═ 11) and a low *FDPS* expression group (*n* ═ 11). The median follow-up duration was 19 months (range, 12–25 months). Survival data were right-censored, with five death events recorded during follow-up. Kaplan–Meier analysis demonstrated that patients with high *FDPS* expression had poorer overall survival than those with low *FDPS* expression, with the difference achieving statistical significance as determined by the log-rank test (*P* ═ 0.0229). The estimated effect size was substantial, with a hazard ratio (HR) of 7.786 and a 95% confidence interval (CI) of 1.223–49.57. However, the broad confidence interval suggests a significant level of uncertainty (Figure 2G).

**Figure 2.**
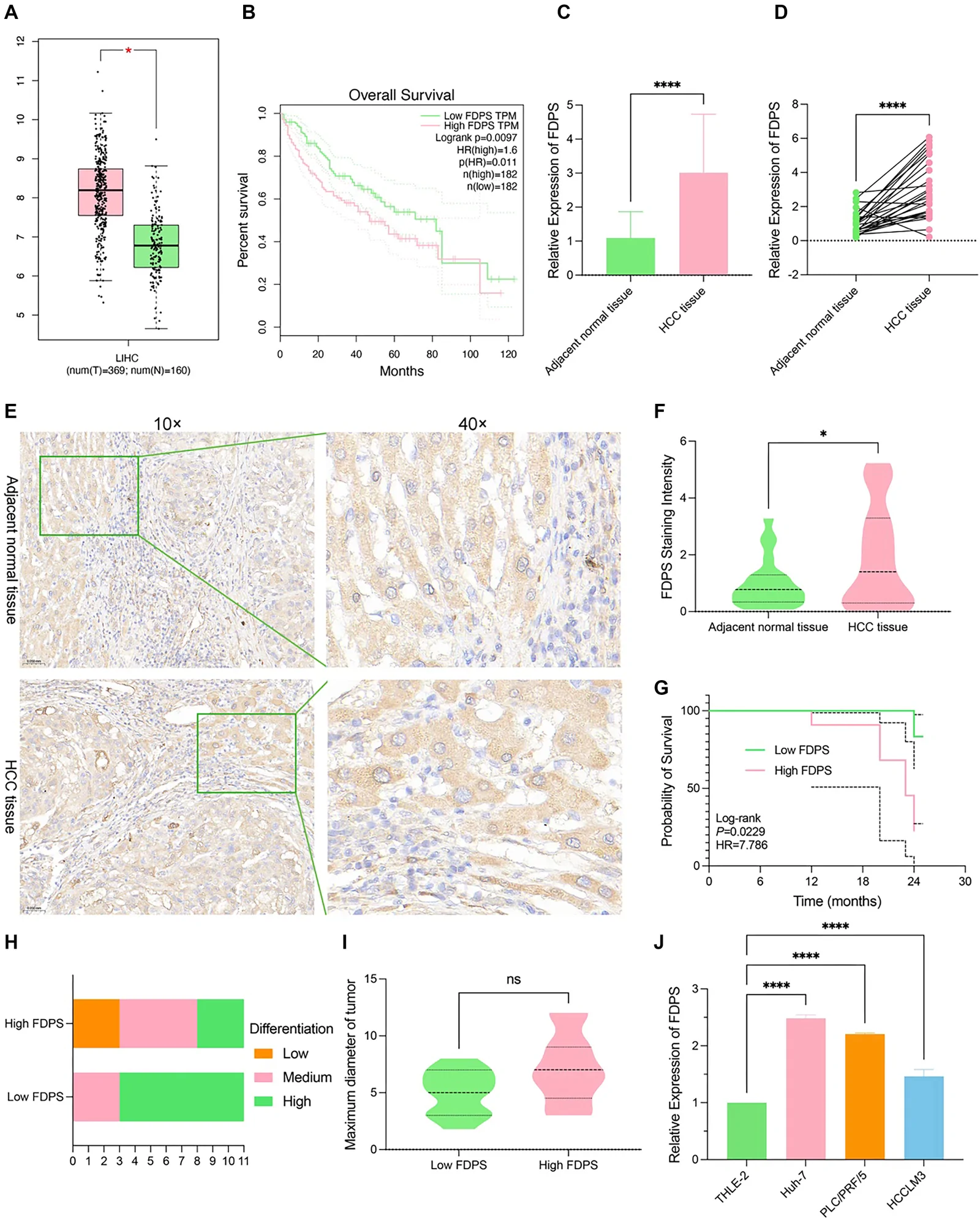
***FDPS* is upregulated in HCC and is potentially associated with poorer survival and adverse clinicopathological features.** (A) GEPIA-based expression analysis showing increased *FDPS* expression in HCC compared with adjacent normal liver tissue. (B) GEPIA overall survival analysis according to *FDPS* expression level. (C) Unpaired qRT-PCR analysis of *FDPS* expression in adjacent normal liver tissues (*n* ═ 30) and HCC tissues (*n* ═ 26). (D) Paired qRT-PCR analysis of *FDPS* expression in 26 matched adjacent normal liver tissue and HCC tissue pairs. (E) Representative immunohistochemical staining of FDPS in adjacent normal liver tissue and HCC tissue at 10× and 40× magnification; boxed areas indicate the regions shown at higher magnification. (F) Quantification of FDPS immunohistochemical staining intensity in adjacent normal liver sections (*n* ═ 21) and HCC sections (*n* ═ 22). (G) Kaplan–Meier overall survival curves for patients stratified by FDPS immunohistochemical expression (low FDPS, n = 11; high FDPS, *n* ═ 11). (H) Distribution of histological differentiation grades in the low- and high-*FDPS* groups. (I) Comparison of maximum tumor diameter between the low- and high-*FDPS* groups. (J) Relative *FDPS* expression in the normal liver cell line THLE-2 and HCC cell lines Huh-7, PLC/PRF/5, and HCCLM3. *P < 0.05; ****P < 0.0001. **Abbreviations:** *FDPS*, farnesyl diphosphate synthase; GEPIA, Gene Expression Profiling Interactive Analysis; HCC, hepatocellular carcinoma; ns, not significant; qRT-PCR, quantitative real-time polymerase chain reaction.

**Figure 3.**
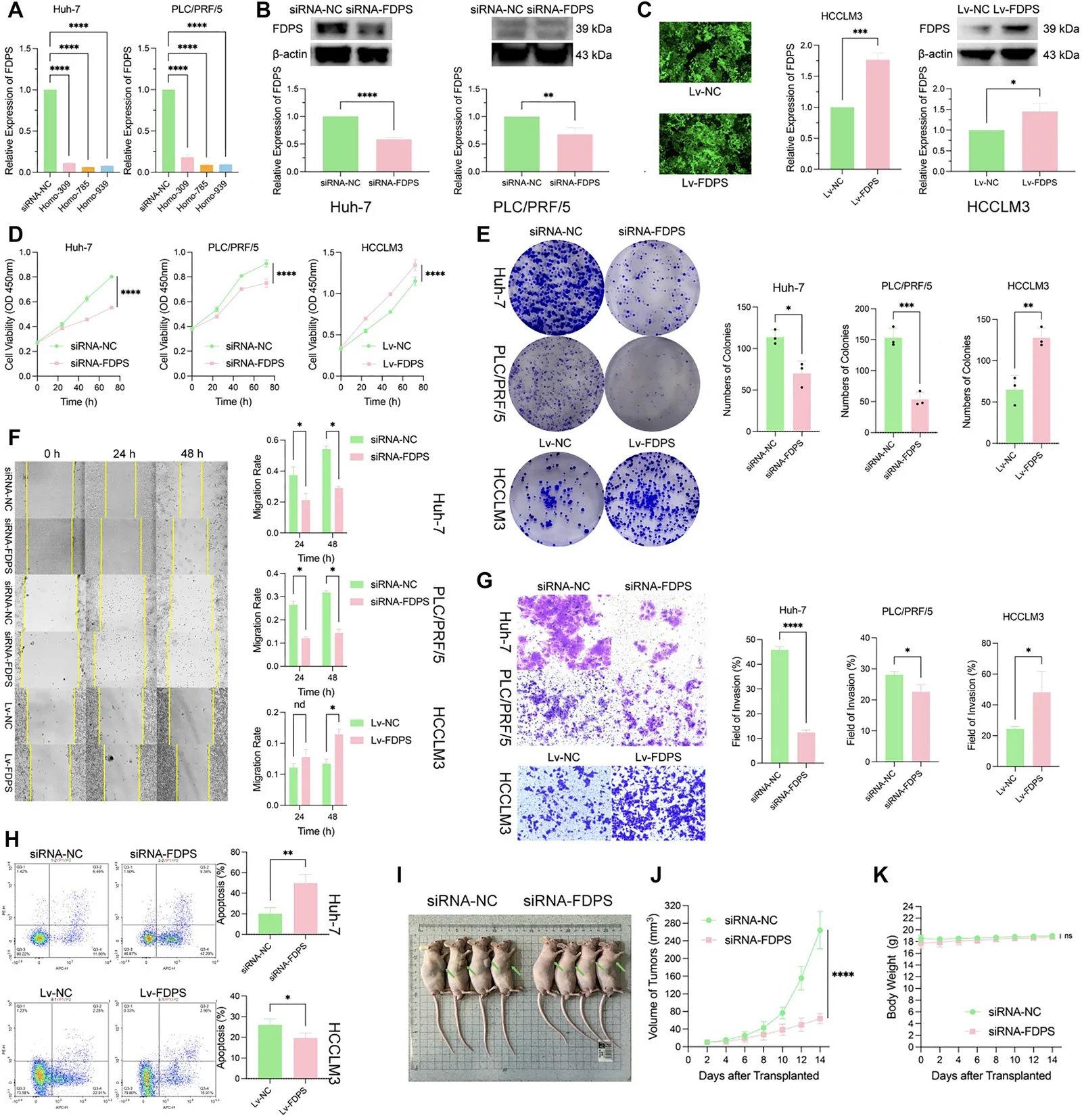
***FDPS* promotes proliferation, migration, invasion, and tumorigenicity while suppressing apoptosis in HCC cells.** (A) qRT-PCR screening of three *FDPS*-targeting small interfering RNAs (Homo-309, Homo-785, and Homo-939) in Huh-7 and PLC/PRF/5 cells, showing efficient reduction of *FDPS* mRNA expression relative to the siRNA-NC group. (B) Western blot analysis and densitometric quantification confirming decreased FDPS protein expression in Huh-7 and PLC/PRF/5 cells after transfection with siRNA-*FDPS* (Homo-785), with β-actin used as the loading control. (C) Representative fluorescence images of lentiviral transduction and qRT-PCR/Western blot validation of *FDPS* overexpression in HCCLM3 cells transduced with Lv-*FDPS* compared with Lv-NC. (D) CCK-8 assays showing that *FDPS* knockdown reduced cell viability in Huh-7 and PLC/PRF/5 cells, whereas *FDPS* overexpression increased cell viability in HCCLM3 cells. (E) Representative colony formation assays and quantification of colony numbers, demonstrating reduced clonogenic growth after *FDPS* knockdown and enhanced clonogenic growth after *FDPS* overexpression. (F) Representative wound-healing images obtained at 0, 24, and 48 h and corresponding migration-rate quantification, showing reduced migratory capacity in *FDPS*-silenced Huh-7 and PLC/PRF/5 cells and increased migration in *FDPS*-overexpressing HCCLM3 cells. (G) Representative Transwell invasion images and quantitative analysis showing that *FDPS* knockdown suppressed invasion in Huh-7 and PLC/PRF/5 cells, whereas *FDPS* overexpression enhanced invasion in HCCLM3 cells. (H) Representative Annexin V-APC/PI flow cytometry plots and apoptosis quantification, showing increased apoptosis after *FDPS* knockdown in Huh-7 cells and reduced apoptosis after *FDPS* overexpression in HCCLM3 cells. (I) Representative images of subcutaneous xenografts derived from Huh-7 cells in the siRNA-NC and siRNA-*FDPS* groups. (J) Xenograft growth curves showing reduced tumor growth in the siRNA-*FDPS* group (*n* ═ 8 per group). (K) Body weight monitoring of tumor-bearing nude mice showing no significant difference between groups during the experimental period (*n* ═ 8 per group). *P < 0.05; **P < 0.01; ***P < 0.001; ****P < 0.0001. **Abbreviations:** APC, allophycocyanin; CCK-8, Cell Counting Kit-8; *FDPS*, farnesyl diphosphate synthase; HCC, hepatocellular carcinoma; Lv-*FDPS*, lentiviral vector encoding *FDPS*; Lv-NC, lentiviral negative control vector; nd, no significant difference; ns, not significant; PI, propidium iodide; qRT-PCR, quantitative real-time polymerase chain reaction; siRNA-*FDPS*, small interfering RNA targeting *FDPS*; siRNA-NC, negative control small interfering RNA.

When *FDPS* expression was modeled as a continuous variable in the Cox proportional hazards model, higher expression was associated with an increased risk of death (HR = 1.427), although this association did not reach statistical significance (95% CI: 0.916–2.221, *P* ═ 0.116). After adjustment for age and sex, the direction of the association remained consistent, although the effect estimate was slightly attenuated and remained statistically non-significant (HR = 1.33, 95% CI: 0.84–2.10, *P* ═ 0.225). Moreover, tumors in the high-*FDPS* group tended to be moderately to poorly differentiated, while those in the low-*FDPS* group were more likely to be moderately to well differentiated (Figure 2H). Additionally, the mean maximum tumor diameter was smaller in the low-*FDPS* group than in the high-*FDPS* group (5.027 ± 1.987 vs. 7.163 ± 3.121), although this difference did not reach statistical significance (*P* ═ 0.0739) (Figure 2I). These findings suggest that *FDPS* is upregulated in HCC and is potentially associated with unfavorable patient outcomes. Compared to the normal hepatic cell line THLE-2, *FDPS* expression was markedly higher in Huh-7 and PLC/PRF/5 cells and slightly higher in HCCLM3 cells (Figure 2J). Huh-7, PLC/PRF/5, and HCCLM3 cell lines were selected for further investigation.

### *FDPS* is associated with enhanced proliferation, migration, invasion and tumorigenicity and reduced apoptosis in HCC cells

To knock down *FDPS* expression in Huh-7 and PLC/PRF/5 cells, siRNAs targeting *FDPS* (siRNA-*FDPS*; Homo-309, Homo-785, and Homo-939) were transfected, with cells transfected with siRNA-NC serving as the negative control (Figure 3A). All three siRNA-*FDPS* sequences produced consistent phenotypic effects in HCC cells (Figure S3). Among the tested sequences, Homo-785 demonstrated the highest *FDPS* knockdown efficiency and was selected for subsequent experiments. Western blot results confirmed that transfection with Homo-785 significantly reduced *FDPS* levels in Huh-7 and PLC/PRF/5 cells (Figure 3B). Conversely, *FDPS* overexpression was induced by lentiviral transduction in HCCLM3 cells, with cells transfected with lentiviral negative control (Lv-NC) serving as the negative control. Fluorescence microscopy confirmed successful lentiviral transduction, with no significant differences in transduction efficiency between the experimental and control groups. Consistent results from qRT-PCR and Western blot analyses demonstrated a significant upregulation of *FDPS* expression in HCCLM3 cells after lentiviral transduction (Figure 3C).

Functionally, CCK-8 and colony formation assays indicated that suppression of *FDPS* was associated with reduced proliferative capacity of HCC cells, while *FDPS* overexpression correlated with increased cell proliferation (Figure 3D and E). Wound healing assays revealed that siRNA-mediated knockdown of *FDPS* diminished the migratory ability of HCC cell lines. Similarly, Transwell assays demonstrated that genetic inhibition of *FDPS* reduced the invasive capacity of Huh-7 and PLC/PRF/5 cells. Conversely, *FDPS* overexpression was associated with enhanced migratory and invasive behaviors in HCCLM3 cells (Figure 3F and G). Annexin V-APC/PI staining combined with flow cytometry indicated that *FDPS* knockdown was associated with increased apoptosis in Huh-7 and PLC/PRF/5 cells, predominantly elevating early apoptotic populations. In contrast, *FDPS* overexpression was linked to reduced early apoptosis in HCCLM3 cells (Figure 3H). Xenograft tumor models were established by subcutaneous injection of *FDPS*-knockdown Huh-7 cells and negative control Huh-7 cells into nude mice, followed by intertumoral administration of siRNA on days 5 and 10 to maintain gene silencing efficiency; FDPS expression in tumor tissues on day 15 is presented in Figure S4. No animals were excluded from the experiments, and each group consisted of eight nude mice. Compared to the control group, knockdown of *FDPS* resulted in reduced tumor growth (Figure 3I and J). Throughout the experimental period, no statistically significant differences in body weight were observed between the two groups (Figure 3K). Collectively, these findings suggest that *FDPS* is associated with enhanced proliferation, migration, invasion, and tumorigenicity, along with reduced apoptosis in HCC cells.

### Pamidronate suppresses proliferation, migration, invasion and tumorigenicity of HCC cells

Pamidronate is one of the most widely utilized N-BPs in clinical practice, serving as a specific inhibitor of FDPS and demonstrating favorable therapeutic efficacy for osteoporosis. Cell Counting Kit-8 (CCK-8) assays revealed that pamidronate significantly inhibited the viability of Huh-7 cells in a dose-dependent manner (Figure 4A). A concentration of 50 µM was selected for subsequent analyses, and ELISA results indicated that this dosage significantly reduced intracellular FPP levels in both Huh-7 and PLC/PRF/5 cells (Figure 4B).

**Figure 4.**
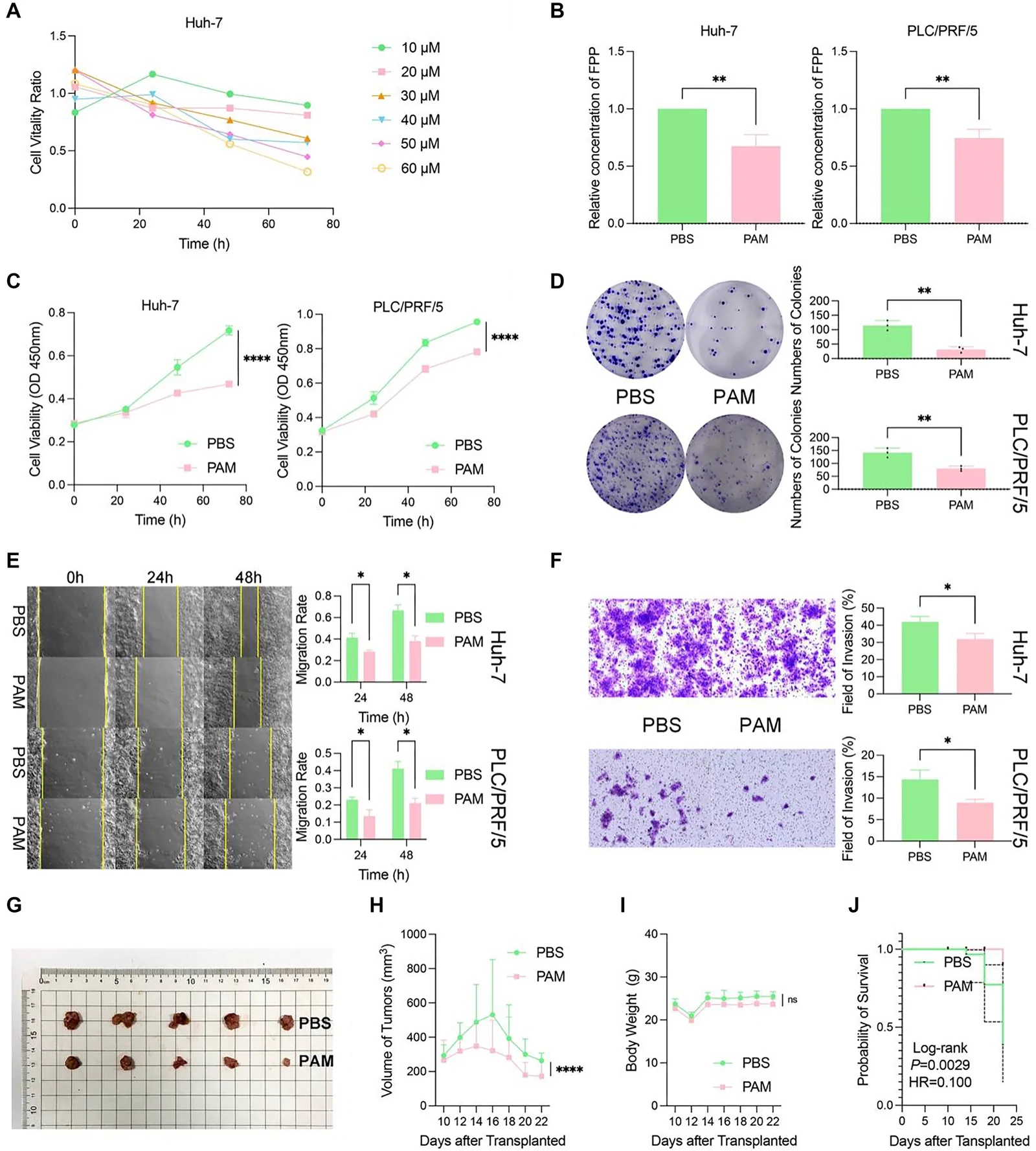
**Pharmacological inhibition of FDPS by pamidronate suppresses proliferation, migration, invasion, and tumorigenicity of HCC cells.** (A) Dose-dependent effect of pamidronate on Huh-7 cell viability over 72 h, supporting the selection of 50 µM for subsequent *in vitro* experiments. (B) Relative intracellular FPP levels in Huh-7 and PLC/PRF/5 cells after treatment with 50 µM pamidronate or PBS. (C) CCK-8 assays showing reduced viability of Huh-7 and PLC/PRF/5 cells following pamidronate treatment. (D) Representative colony formation assays and quantification of colony numbers demonstrating decreased clonogenic growth after pamidronate exposure. (E) Representative wound-healing images acquired at 0, 24, and 48 h and corresponding migration-rate quantification, showing impaired migratory capacity in pamidronate-treated Huh-7 and PLC/PRF/5 cells. (F) Representative Transwell invasion images and quantitative analysis showing reduced invasive capacity after pamidronate treatment. (G) Representative subcutaneous tumors excised from C57BL/6 mice bearing Hepa1-6 tumors treated with pamidronate or PBS. (H) Tumor growth curves showing that intraperitoneal pamidronate administration suppressed tumor growth *in vivo*. (I) Body weight monitoring of tumor-bearing mice showing no significant difference between groups. (J) Kaplan–Meier survival curves showing improved survival in pamidronate-treated tumor-bearing mice. *P < 0.05; **P < 0.01; ****P < 0.0001. **Abbreviations:** CCK-8, Cell Counting Kit-8; FDPS, farnesyl diphosphate synthase; FPP, farnesyl pyrophosphate; HCC, hepatocellular carcinoma; ns, not significant; PAM, pamidronate; PBS, phosphate-buffered saline.

Consistent with the effects observed following genetic inhibition of *FDPS*, pamidronate treatment effectively suppressed the proliferation, migration, and invasion of HCC cells (Figure 4C–F). C57BL/6 mice were subcutaneously injected with Hepa1-6 cells to establish transplanted tumor models. All animals were included in the analyses, with each group comprising ten mice. Compared to the control group, intraperitoneal administration of pamidronate significantly inhibited tumor growth (Figure 4G and H). Throughout the experimental period, no significant differences in body weight were observed between the two groups of mice (Figure 4I). Furthermore, treatment with pamidronate improved the survival of tumor-bearing mice (Figure 4J). Notably, tumor regression was observed in both groups over time, potentially due to the intact and robust anti-tumor immune responses characteristic of the C57BL/6 mouse strain. N-BPs have been reported to exhibit immunomodulatory properties, suggesting that employing immunocompetent mice provides a more physiologically relevant model to better reflect the potential anti-tumor effects of pamidronate *in vivo*. Collectively, these findings indicate that FDPS inhibition suppresses multiple malignant phenotypes of HCC cells and support further investigation of FDPS inhibition as a potential therapeutic strategy for HCC.

### Phosphorylation landscape changes following *FDPS* depletion in HCC cells

Prior studies have suggested a role for *FDPS* in regulating protein phosphorylation. To elucidate the molecular mechanisms associated with *FDPS* in HCC progression, we conducted an LC–MS/MS-based phosphoproteomic analysis to assess phosphorylation changes following *FDPS* depletion in HCC cells. The mass spectrometry data, analyzed using FragPipe software, identified 4,895 phosphoproteins, comprising 20,223 phosphopeptides and 25,347 phosphorylation sites (Figure 5A). Quality assessment revealed adequate peptide abundance across all samples, with no significant differences between the experimental and control groups (Figure 5B). Correlation analysis indicated that phosphorylation profiles in the siRNA-*FDPS* group were more homogeneous and distinct from those in the siRNA-NC group (Figure 5C). Principal component analysis further demonstrated distinct phosphorylation patterns between *FDPS*-depleted and control HCC cells (Figure 5D). Among all differentially phosphorylated sites, 615 exhibited decreased phosphorylation levels following *FDPS* downregulation, while 1,977 showed increased phosphorylation levels (Figure 5E). These differentially phosphorylated sites were predominantly located in the nucleus, cytoplasm, plasma membrane, and mitochondria (Figure 5F). Conserved motif analysis revealed that the (...TPP...) motif was the most highly enriched among the amino acids surrounding the phosphorylated sites (Figure 5G).

**Figure 5.**
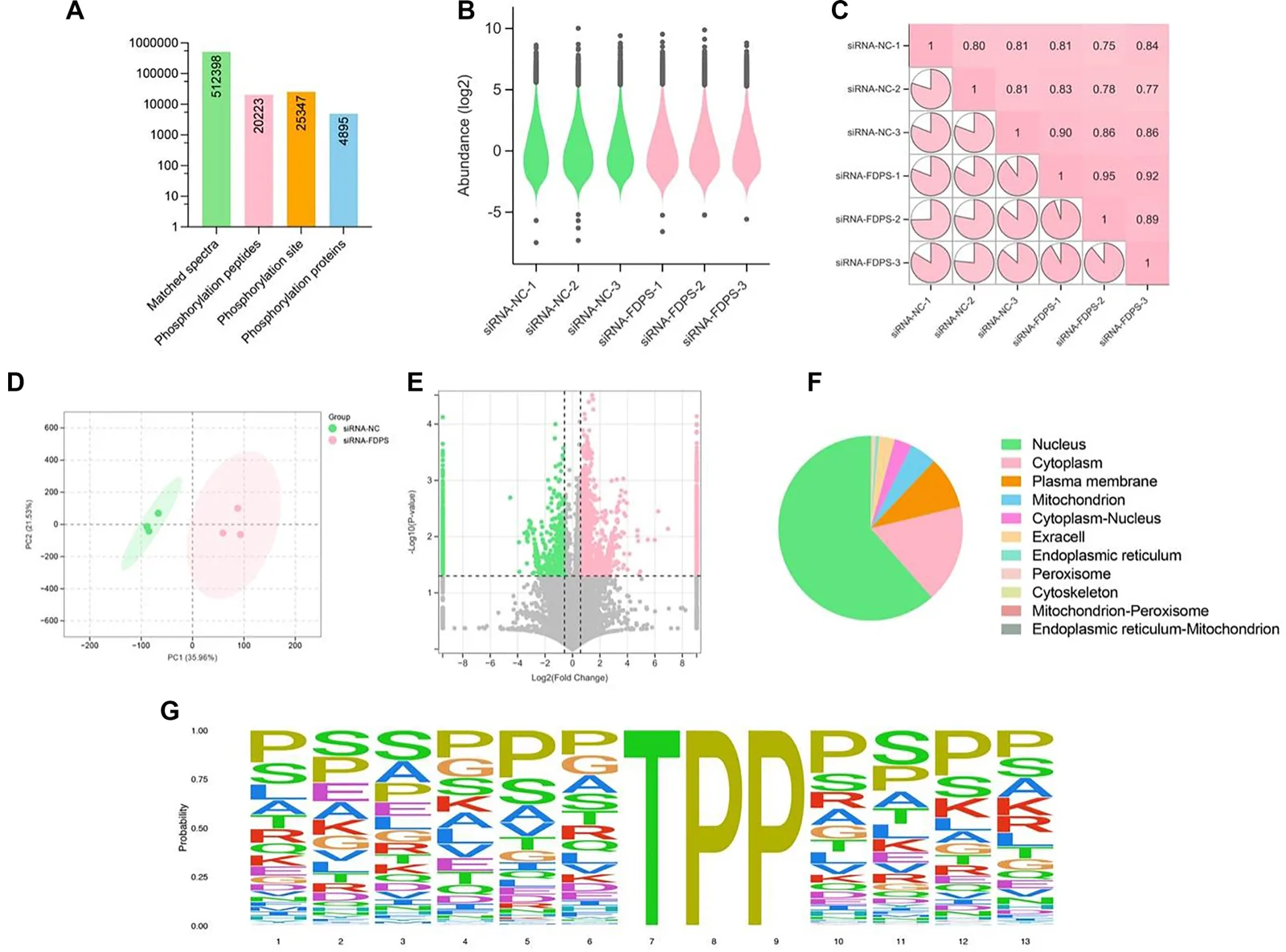
**LC–MS/MS-based phosphoproteomic profiling reveals widespread phosphorylation changes after *FDPS* depletion in Huh-7 cells.** (A) Summary of phosphoproteomic identification results, showing the numbers of matched spectra, phosphopeptides, phosphorylation sites, and phosphoproteins identified in Huh-7 cells. (B) Violin plots showing the distribution of phosphopeptide abundance across three siRNA-NC and three siRNA-*FDPS* samples, indicating adequate peptide abundance and overall comparability among samples. (C) Inter-sample correlation matrix demonstrating high reproducibility within each group and clear differences between control and *FDPS*-silenced samples. (D) Principal component analysis of phosphoproteomic profiles showing distinct clustering of the siRNA-NC and siRNA-*FDPS* groups. (E) Volcano plot of differentially phosphorylated sites following *FDPS* knockdown; candidate sites were defined using *P* < 0.05 and |log2 fold change| > 0.58, with 615 sites showing decreased phosphorylation and 1,977 sites showing increased phosphorylation relative to the control group. (F) Subcellular localization of differentially phosphorylated proteins, showing predominant distribution in the nucleus, cytoplasm, plasma membrane, and mitochondrion. (G) Sequence logo of the most highly enriched conserved phosphorylation motif derived from amino acid residues flanking the modified sites, with the (...TPP...) motif showing the strongest enrichment. **Abbreviations:** *FDPS*, farnesyl diphosphate synthase; HCC, hepatocellular carcinoma; LC–MS/MS, liquid chromatography–tandem mass spectrometry; PCA, principal component analysis; siRNA-*FDPS*, small interfering RNA targeting *FDPS*; siRNA-NC, negative control small interfering RNA.

**Figure 6.**
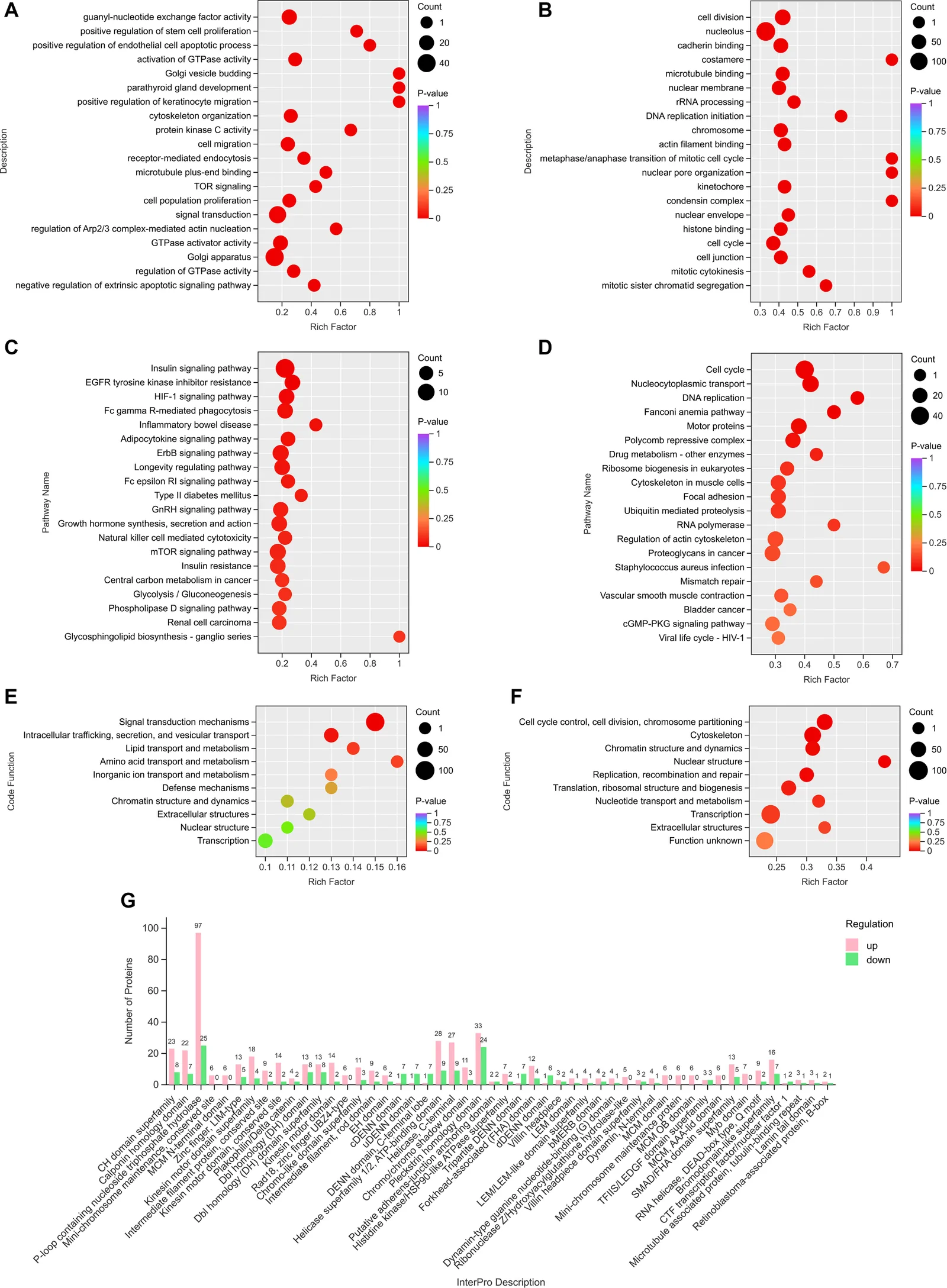
**Exploratory enrichment analyses of differentially phosphorylated proteins associated with *FDPS* depletion in Huh-7 cells.** (A) GO enrichment analysis of proteins harboring phosphosites with decreased phosphorylation abundance after *FDPS* knockdown, suggesting potential associations with cell proliferation, migration, cytoskeletal organization, protein kinase C activity, GTPase regulation, Golgi apparatus function, endocytosis, and apoptosis-related processes. (B) GO enrichment analysis of proteins harboring phosphosites with increased phosphorylation abundance after *FDPS* knockdown, showing enrichment in cell cycle progression, DNA replication, mitotic processes, and nuclear structural organization. (C) KEGG enrichment analysis of proteins with decreased phosphorylation abundance, indicating potential involvement of glucose metabolism, tumor immune regulation, mechanistic target of rapamycin signaling, and other cancer-related pathways. (D) KEGG enrichment analysis of proteins with increased phosphorylation abundance, highlighting pathways associated with cell cycle regulation, nucleocytoplasmic transport, DNA replication and repair, cytoskeletal regulation, and post-transcriptional processes. (E) KOG annotation of proteins with decreased phosphorylation abundance, showing enrichment in signal transduction, intracellular trafficking and vesicular transport, lipid transport and metabolism, chromatin-related functions, and transcription. (F) KOG annotation of proteins with increased phosphorylation abundance, showing enrichment in cell cycle control, chromosome partitioning, cytoskeleton, replication and repair, translation, and nucleotide transport and metabolism. (G) InterPro domain enrichment analysis of differentially phosphorylated proteins, showing enrichment of calponin homology domain-containing proteins and P-loop-containing nucleoside triphosphate hydrolases, suggesting possible involvement of cytoskeletal remodeling and nucleotide-dependent regulatory proteins. In panels A–F, bubble size indicates protein count, color indicates *P* value, and the x-axis indicates the Rich Factor. In panel G, pink and green bars denote domains enriched among proteins with increased and decreased phosphorylation abundance, respectively. **Abbreviations:** *FDPS*, farnesyl diphosphate synthase; GO, Gene Ontology; KEGG, Kyoto Encyclopedia of Genes and Genomes; KOG, euKaryotic Orthologous Groups.

GO enrichment analysis indicated that *FDPS* depletion was associated with decreased phosphorylation of proteins involved in cell proliferation, migration, invasion, cancer stem cell maintenance, and protein kinase C activity. These findings suggest a potential link between *FDPS* and the phosphorylation-dependent regulation of malignant HCC phenotypes. Additionally, phosphorylation attenuation associated with extrinsic apoptosis was observed following *FDPS* depletion, indicating a possible involvement of *FDPS* in apoptosis-related processes. Phosphorylation alterations were also enriched in pathways related to GTPase activity, mTOR signaling, Golgi apparatus function, and endocytic processes (Figure 6A). *FDPS* depletion correlated with increased phosphorylation of cell cycle-related proteins, suggesting that *FDPS* may negatively regulate phosphorylation in cell cycle-related processes. Furthermore, these phosphorylation changes may be linked to post-transcriptional regulatory mechanisms (Figure 6B). KEGG pathway analysis demonstrated that the decrease in phosphorylation associated with *FDPS* depletion was enriched in pathways related to glucose metabolism and tumor immune regulation in HCC cells. These alterations were also mapped to several cancer-related signaling pathways, including hypoxia-inducible factor 1 (HIF-1), ErbB, gonadotropin-releasing hormone (GnRH), and phospholipase D signaling (Figure 6C). In alignment with the GO enrichment results, KEGG pathway analysis revealed that the increase in phosphorylation associated with *FDPS* depletion was enriched in pathways related to the cell cycle and post-transcriptional regulation (Figure 6D). Consistent with the GO enrichment results, euKaryotic Orthologous Groups (KOG) annotation indicated that the reduction in phosphorylation associated with *FDPS* depletion was enriched in functional categories related to Golgi-associated intracellular transport, signal transduction, and lipid metabolism (Figure 6E). Additionally, refined KOG annotation suggested that *FDPS* may influence cell cycle regulation and protein expression in HCC through the suppression of phosphorylation (Figure 6F). InterPro analysis revealed the enrichment of calponin homology (CH) domain-containing proteins, typically associated with actin binding and cytoskeletal organization. Enrichment of P-loop containing nucleoside triphosphate hydrolases was also observed, suggesting a potential association between adenosine triphosphate (ATP)- and guanosine triphosphate (GTP)-dependent regulatory proteins and the observed phosphorylation changes (Figure 6G). In summary, *FDPS* depletion is associated with widespread changes in protein phosphorylation in HCC cells, highlighting a potential role for *FDPS* in phosphorylation-related regulatory processes relevant to HCC progression.

### *FDPS* is associated with changes in Bcl-2, Caspase-9/3, and mTOR signaling in HCC cells

In this study, genetic suppression of *FDPS* significantly increased apoptosis in HCC cells, whereas treatment with pamidronate did not markedly affect apoptosis (Figure S5). These observations suggest that the regulatory effect of FDPS on apoptosis may not be mediated by its metabolic product, FPP. Moreover, phosphoproteomic analysis indicated a potential involvement of *FDPS* in the regulation of apoptotic pathways in HCC cells. Consequently, Western blot analysis was performed to assess the expression and phosphorylation levels of key apoptosis-related proteins in HCC cells following *FDPS* depletion. The results indicated that *FDPS* knockdown was associated with decreased levels of Bcl-2, an anti-apoptotic protein, and phosphorylated Caspase-9 (Ser196), a modification linked to reduced caspase activity, along with increased levels of cleaved Caspase-3, a marker of apoptosis activation, in HCC cells. These findings suggest a potential role for *FDPS* in apoptosis-related processes, possibly linked to alterations in Bcl-2 expression and Caspase-9 phosphorylation (Figure 7A). However, the mechanisms by which *FDPS* regulates apoptosis in HCC cells via Bcl-2 and Caspase-9/3 warrant further investigation.

**Figure 7.**
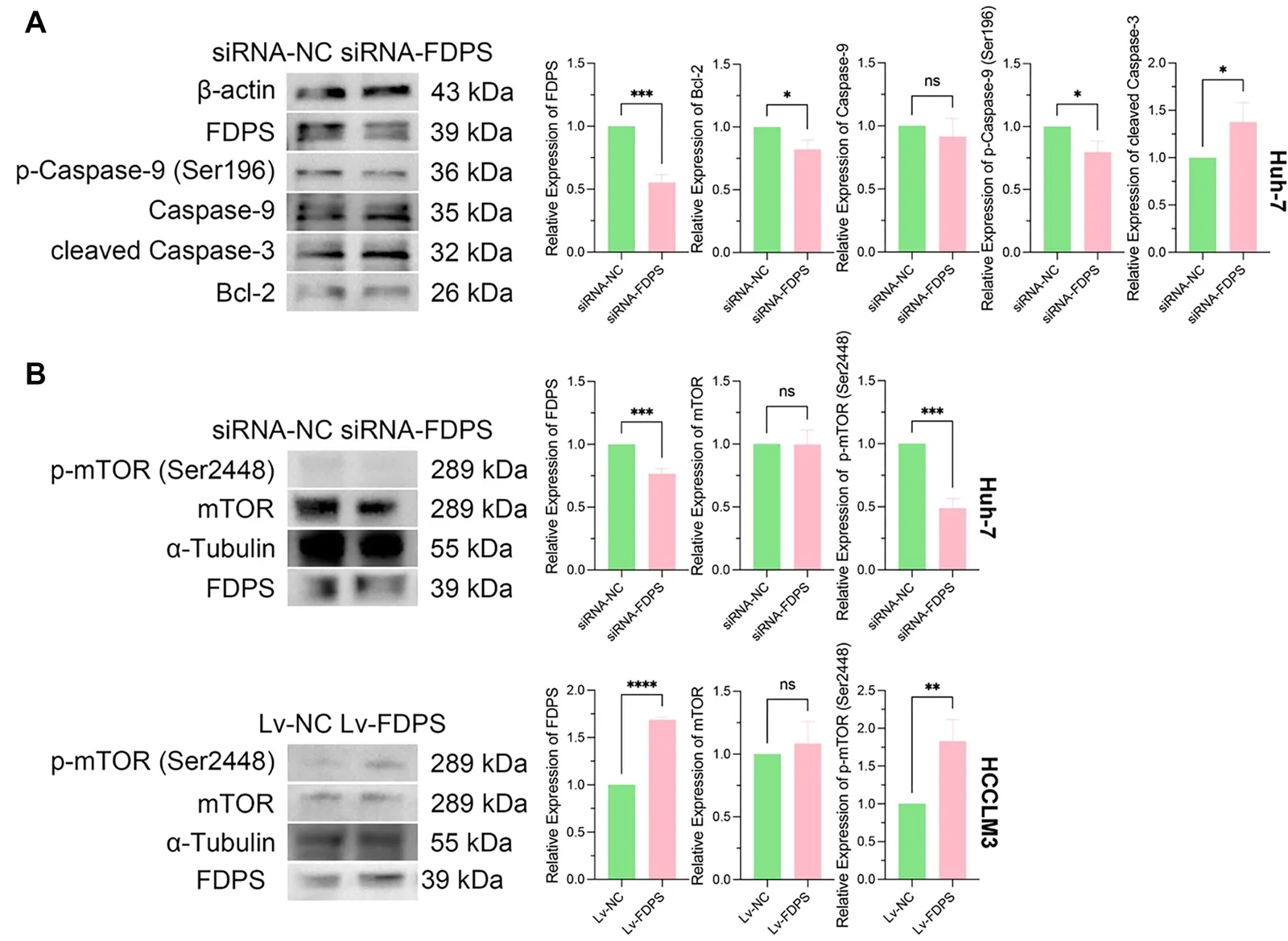
***FDPS* modulation is associated with changes in apoptosis-related proteins and mTOR phosphorylation in HCC cells.** (A) Representative Western blots and densitometric quantification in Huh-7 cells transfected with siRNA-NC or siRNA-*FDPS*, showing that *FDPS* knockdown was associated with reduced Bcl-2 expression, decreased phosphorylation of Caspase-9 at Ser196, increased cleaved Caspase-3 expression, and no significant change in total Caspase-9 levels. β-actin was used as the loading control. (B) Representative Western blots and densitometric quantification of mTOR signaling after *FDPS* modulation. In Huh-7 cells, *FDPS* knockdown was associated with reduced phosphorylation of mTOR at Ser2448 without a significant change in total mTOR expression. In HCCLM3 cells, lentiviral *FDPS* overexpression was associated with increased phosphorylation of mTOR at Ser2448, whereas total mTOR levels remained unchanged. α-Tubulin was used as the loading control. *P < 0.05; **P < 0.01; ***P < 0.001; ****P < 0.0001. **Abbreviations:** Bcl-2, B-cell lymphoma 2; *FDPS*, farnesyl diphosphate synthase; HCC, hepatocellular carcinoma; Lv-*FDPS*, lentiviral vector encoding *FDPS*; Lv-NC, lentiviral negative control vector; mTOR, mechanistic target of rapamycin; ns, not significant; siRNA-*FDPS*, small interfering RNA targeting *FDPS*; siRNA-NC, negative control small interfering RNA.

**Figure 8.**
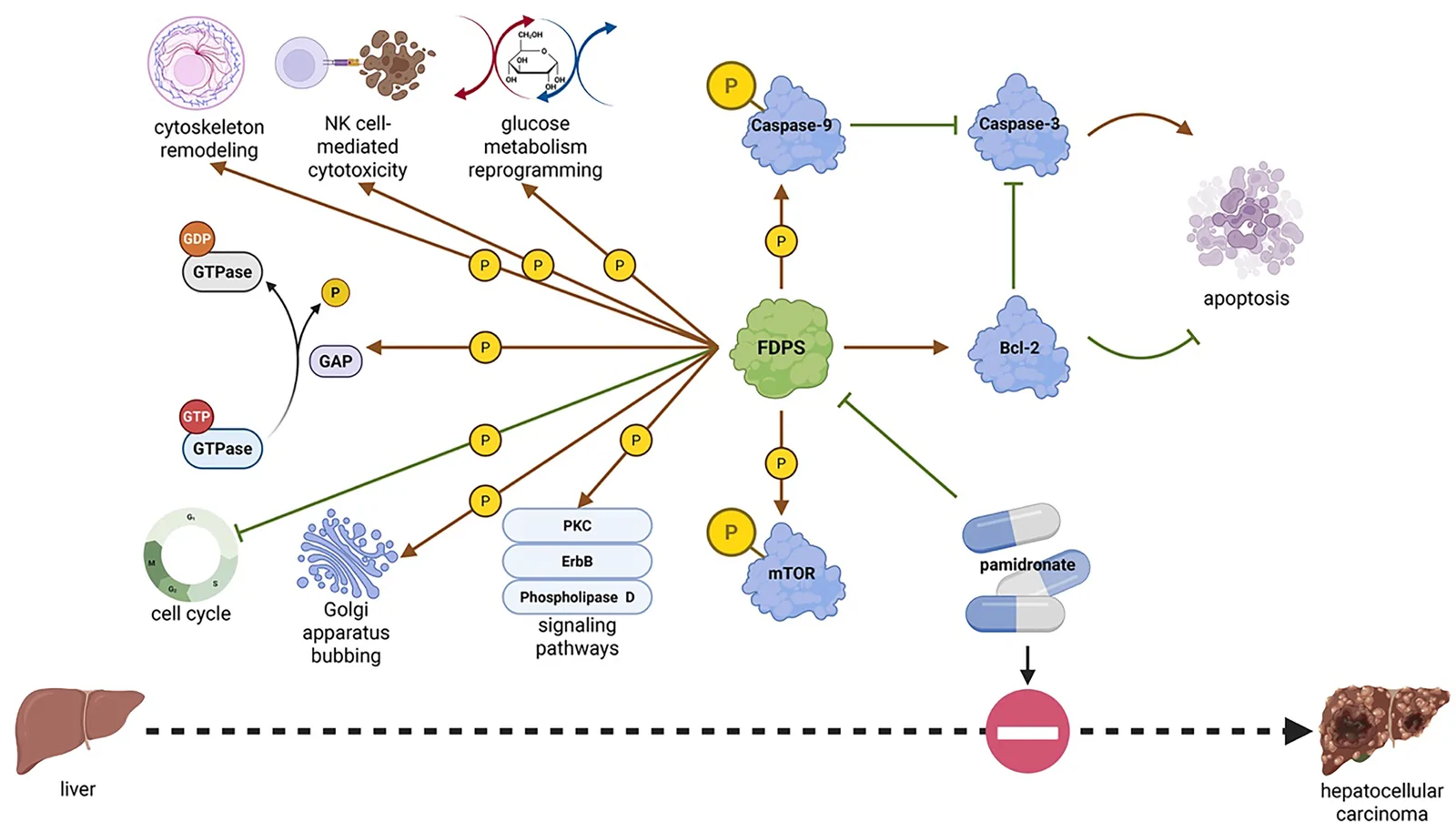
**Proposed *FDPS*-associated regulatory network in HCC based on functional and phosphoproteomic analyses.** This schematic summarizes the putative mechanisms by which *FDPS* may contribute to HCC progression. Elevated *FDPS* expression is proposed to promote phosphorylation-associated regulatory programs linked to GTPase activity and GTPase-activating proteins, protein kinase C, ErbB and phospholipase D signaling, glucose metabolism reprogramming, cytoskeletal remodeling, Golgi apparatus function, natural killer cell-mediated cytotoxicity, and cell cycle-related processes. In parallel, *FDPS* may suppress apoptosis by increasing Bcl-2 expression and promoting phosphorylation of Caspase-9, thereby limiting Caspase-3 activation, and may also be associated with increased phosphorylation of mTOR. Pamidronate is shown as a pharmacological inhibitor of *FDPS*, supporting the concept that FDPS targeting may attenuate HCC progression. Yellow circles marked “P” indicate phosphorylation events; arrows indicate proposed positive regulatory associations, whereas blunt-ended lines indicate inhibition. **Abbreviations:** Bcl-2, B-cell lymphoma 2; *FDPS*, farnesyl diphosphate synthase; GAP, GTPase-activating protein; GDP, guanosine diphosphate; GTP, guanosine triphosphate; HCC, hepatocellular carcinoma; mTOR, mechanistic target of rapamycin; NK, natural killer; PKC, protein kinase C.

Consistent with the phosphoproteomic data, Western blot analysis demonstrated that *FDPS* knockdown was associated with reduced phosphorylation of mTOR at Ser2448, a site commonly associated with mTOR signaling activity, in Huh-7 cells, while *FDPS* overexpression corresponded with increased phosphorylation at this site in HCCLM3 cells. These findings indicate a potential association between *FDPS* and mTOR signaling activity in HCC cells (Figure 7B).

## Discussion

In this study, we systematically examined the expression patterns, functional relevance, and therapeutic responsiveness of *FDPS* in HCC, along with an initial exploration of the underlying molecular mechanisms. Analysis of public datasets and clinical samples revealed elevated *FDPS* expression in HCC tissues compared to adjacent normal tissues. A potential association between higher *FDPS* expression and poorer survival outcomes was suggested; however, the evidence remains inconclusive due to inconsistencies in analytical approaches and imprecision in effect estimates. Further validation is warranted.

Functional assays demonstrated that siRNA-mediated knockdown of *FDPS* resulted in decreased proliferation, migration, invasion, and tumorigenic capacity of HCC cells, accompanied by increased apoptosis. Conversely, *FDPS* overexpression induced opposite phenotypic changes. Additionally, pamidronate treatment exhibited inhibitory effects on HCC cells comparable to those resulting from FDPS suppression. Collectively, these findings suggest that *FDPS* may contribute to the malignant phenotype of HCC and highlight its potential as a biomarker and therapeutic target. Given the established clinical application of N-BPs in osteoporosis, targeting FDPS with these agents may represent a promising therapeutic strategy for HCC, warranting further investigation.

LC-MS/MS-based phosphoproteomic analysis identified widespread alterations in protein phosphorylation following *FDPS* depletion in Huh-7 cells, suggesting a possible association between *FDPS* and phosphorylation-related biological processes. We identified 615 phosphorylation sites with increased abundance and 1,977 sites with decreased abundance following *FDPS* depletion. Proteins containing these phosphorylation sites were predominantly localized to the nucleus, cytoplasm, plasma membrane, and mitochondria. Enrichment analyses indicated that decreases in phosphorylation following *FDPS* depletion were associated with HCC cell proliferation, migration, invasion, and cancer stem cell-related functions. These pro-tumorigenic effects may be mediated by cytoskeletal remodeling and the activation of the protein kinase C family [21–23]. Phosphoproteomic analysis further suggested that reductions in phosphorylation associated with *FDPS* depletion were enriched in pathways related to GTPase regulation. Small GTPases are widely implicated in various cellular processes, including proliferation, metabolism, senescence, migration, and stemness, as well as in regulating the tumor microenvironment and inflammatory responses [24, 25]. These observations suggest a potential link between *FDPS*-associated phosphorylation changes and GTPase-mediated HCC progression. Furthermore, phosphoproteomic analysis indicated that *FDPS* may enhance the phosphorylation abundance of Golgi-associated proteins, including Golgi phosphoprotein 3 (GOLPH3), an oncoprotein in HCC known to activate mTOR signaling [26]. Phosphorylation changes associated with *FDPS* were also enriched in pathways related to endocytic processes and natural killer cell-mediated cytotoxicity, underscoring their potential relevance to therapeutic targeting strategies in HCC [27, 28]. KEGG pathway enrichment analyses further suggested that *FDPS* may contribute to the reprogramming of glucose metabolism by inducing phosphorylation in HCC cells [29]. Among the cancer-related pathways potentially affected by *FDPS*-associated phosphorylation, HIF-1 and ErbB signaling have been widely reported to play important roles in HCC [30, 31]. Additionally, phosphorylation sites exhibiting increased abundance following *FDPS* depletion were enriched in processes related to DNA replication, sister chromatid segregation, and cell division, as well as components such as the condensin complex and nuclear pore organization. These findings suggest a potential association between *FDPS*-related phosphorylation reduction and cell cycle regulation in HCC [32, 33]. However, these results are preliminary and exploratory, requiring validation in independent studies.

While genetic depletion of *FDPS* enhanced apoptosis in HCC cells, pharmacological inhibition of FDPS using pamidronate did not yield a comparable effect. This discrepancy implies that the effects of *FDPS* on apoptosis may be independent of its metabolic catalytic properties. To further explore the potential links between *FDPS* and apoptosis, we examined the effects of *FDPS* knockdown on the expression and phosphorylation of key apoptosis-related proteins. Western blot analysis revealed that *FDPS* knockdown was associated with decreased Bcl-2 expression and reduced phosphorylation of Caspase-9 at Ser196, accompanied by increased levels of cleaved Caspase-3 in HCC cells. Bcl-2 is recognized as a key regulator of apoptosis, capable of inhibiting cell death by elevating the mitochondrial apoptotic threshold [34]. Phosphorylation of Caspase-9 at Ser196 could suppress its activity, thereby blocking the cleavage and activation of the downstream executioner Caspase-3 and inhibiting programmed cell death [35, 36]. Moreover, Bcl-2 may inhibit mitochondrial cytochrome c release, suppressing downstream Caspase-3 activation in the apoptotic pathway [37]. Collectively, these findings suggest that *FDPS* may impede apoptosis in HCC cells by inducing Bcl-2 expression and phosphorylation-mediated inactivation of Caspase-9, thereby suppressing the cleavage and activation of Caspase-3. These results should be considered as a hypothesis, requiring further validation in future studies.

Activation of mTOR signaling is recognized as a promoter of tumor growth and metastasis [38, 39]. Phosphorylation of mTOR at Ser2448, a commonly used indicator of mechanistic target of rapamycin complex 1 (mTORC1) activity, reflects the activation status of mTOR signaling [40–42]. In our study, phosphorylation of mTOR at Ser2448 was reduced following *FDPS* knockdown and elevated upon *FDPS* overexpression, suggesting a potential association between *FDPS* expression and mTOR signaling activity. The pathway by which *FDPS* mediates mTOR signaling activation warrants further investigation.

In summary, *FDPS* expression was elevated in HCC and was potentially associated with unfavorable prognosis. Functional assays indicated that *FDPS* is linked to HCC cell proliferation, migration, invasion, and tumorigenicity, while being associated with reduced apoptosis. Phosphoproteomic analysis demonstrated that *FDPS* depletion was accompanied by widespread changes in protein phosphorylation. Exploratory enrichment analyses further suggested that these phosphorylation changes may be associated with the activation of GTPase and protein kinase C, glucose metabolism, cytoskeletal organization, immune-related processes, cell cycle regulation, Golgi apparatus function, and cancer-related signaling pathways such as ErbB and phospholipase D (Figure 8).

Importantly, further experimental validation indicated that *FDPS* knockdown was associated with decreased Bcl-2 expression, reduced phosphorylation of Caspase-9 at Ser196, and increased levels of cleaved Caspase-3, suggesting a potential involvement of *FDPS* in the regulation of apoptosis. *FDPS* expression was also associated with changes in mTOR phosphorylation at Ser2448, indicating a possible link between *FDPS* and mTOR signaling activation.

Despite these findings, several limitations of this study should be acknowledged. First, the relatively limited sample size may restrict the generalizability of our observations. Although the findings are supported by consistent siRNA-based loss-of-function and gain-of-function experiments, the absence of siRNA rescue experiments remains a limitation. Moreover, while our data suggest potential mechanisms underlying the role of *FDPS* in HCC, the mechanistic insights remain preliminary and require further validation. Future studies with larger cohorts and more in-depth functional and mechanistic investigations will be necessary to comprehensively elucidate the role of *FDPS* in HCC.

## Conclusion

In conclusion, *FDPS* is highly expressed in HCC tissues and is potentially associated with unfavorable prognosis in patients with HCC. *FDPS* enhances the malignant phenotype of HCC cells and exerts broad effects on the phosphoproteomic landscape. Furthermore, pharmacological inhibition of *FDPS* using N-BPs may offer a potential therapeutic avenue for HCC.

## Supplemental data

Supplemental data are available at the following link: https://www.bjbms.org/ojs/index.php/bjbms/article/view/13996/4173.

## Data Availability

All data can be requested from the corresponding author for appropriate reasons.
